# Research Progress on the Mechanism Between Polycystic Ovary Syndrome and Abnormal Endometrium

**DOI:** 10.3389/fphys.2021.788772

**Published:** 2021-12-17

**Authors:** Zhu Xue, Juanli Li, Jiaxing Feng, Han Han, Jing Zhao, Jiao Zhang, Yanhua Han, Xiaoke Wu, Yuehui Zhang

**Affiliations:** ^1^The graduate school, Heilongjiang University of Chinese Medicine, Harbin, China; ^2^The First Clinical Hospital Affiliated to Harbin Medical University, Harbin, China; ^3^Heilongjiang Academy of Traditional Chinese Medicine, Harbin, China; ^4^Department of Acupuncture and Moxibustion, Second Affiliated Hospital, Heilongjiang University of Chinese Medicine, Harbin, China; ^5^Department of Obstetrics and Gynecology, First Affiliated Hospital, Heilongjiang University of Chinese Medicine, Harbin, China

**Keywords:** polycystic ovary syndrome, endometrium, anovulation, insulin resistance, hyperandrogenemia, progesterone resistance, inflammatory cytokines

## Abstract

As a highly dynamic tissue, the endometrium is periodically shed in response to the secretion of estrogen and progesterone. After menarche, the endometrium of healthy women proliferates and differentiates under the action of steroid hormones (e.g., 17β-estradiol and progesterone) that are secreted by the ovaries to provide appropriate conditions for embryo implantation. Polycystic ovary syndrome (PCOS), a prevalent endocrine and metabolic disorder in reproductive-aged women, is usually associated with multiple cysts within the ovaries and excess levels of androgen and is characterized by hirsutism, acne, menstrual irregularity, infertility, and increased risk of insulin resistance. Multiple factors, such as anovulation, endocrine-metabolic abnormalities, and inflammation, can disrupt the endometrium in PCOS patients and can lead to endometrial hyperplasia, pregnancy complications, or even cancer. Despite many recent studies, the relationship between PCOS and abnormal endometrial function is still not fully understood. In this review, we investigate the correlation of PCOS patient endometrium with anovulation, hyperandrogenemia, insulin resistance, progesterone resistance, and inflammatory cytokines, aiming to provide a theoretical basis for the treatment of disorders caused by endometrial dysfunction in PCOS patients.

## Introduction

Polycystic ovary syndrome (PCOS) is a complex reproductive and metabolic disease that affects 4–21% of adolescent and childbearing women worldwide (Lizneva et al., 2016). Its pathological features include hyperandrogenemia (HA), anovulation, and multiple cysts within the ovaries, and it is often accompanied by obesity and insulin resistance (IR). Women of reproductive age with PCOS usually have reproductive disorders, such as low pregnancy rate, low live birth rate, and high abortion rate. The pregnancy rate of PCOS patients is low even with high-quality embryos, which indicates that apart from anovulation many pathological manifestations may also be related to the endometrial microenvironment (Goodarzi et al., 2011; Li et al., 2016; Cooney and Dokras, 2018). During the menstrual cycle, the endometrium undergoes remodeling, shedding, and regeneration to provide suitable conditions for blastocyst implantation and the establishment of pregnancy, and well-functioning endometrial receptivity is one of the indispensable factors to ensure embryo implantation. Studies have shown that pregnant women with PCOS have 3–4 times higher incidence of gestational hypertension and preeclampsia owing to decidualization/placental changes and a 2-fold higher risk of preterm birth compared to healthy women (Zhao et al., 2021), and this is likely due to endometrial progesterone resistance (Dunk et al., 2019; George et al., 2020; Eisman et al., 2021; Ohara et al., 2021). IR is a common metabolic abnormality in PCOS patients. Compensatory hyperinsulinemia increases the bio-availability of androgen and its production in the ovary and adrenal gland by reducing the concentration of sex hormone-binding globulin, and high levels of androgen and insulin in the plasma can affect the periodic exfoliation of the endometrium (McCartney and Marshall, 2016).

**Figure 1:**
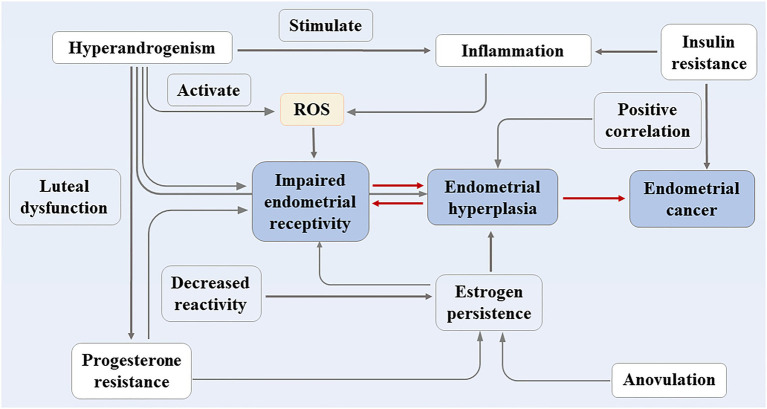
The clinical phenotype of PCOS and the related mechanisms of endometrial physiology and pathology.

For PCOS patients who do not want to become pregnant, the incidence of endometrial carcinoma increases substantially due to the long duration of irregular menstruation. The study conducted by Meczekalski et al. showed that the risk of endometrial carcinoma in PCOS patients is approximately three times higher than that of healthy women (Meczekalski et al., 2020). The endometrium contains various immune cells, which, together with cytokines and chemokines, maintain its function (McCartney and Marshall, 2016), and during menstruation, the immune cells and the inflammatory response of the body fluctuate with hormone changes. The follicular period is dominated by T cells, and the macrophages in the secretory period, especially the uterine natural killer cells (UNKs), increase (Strobel et al., 2021). The abnormal hormone levels in patients with PCOS make the cytokine/chemokine spectrum fluctuate, which also has adverse effects on the endometrial environment. This article reviews the clinical phenotype of PCOS, the related mechanisms of endometrial physiology and pathology (shown in Figure 1 for details) in order to provide a more accurate theoretical basis for its clinical treatment.

## Anovulation and PCOS Endometrium

Polycystic ovary syndrome patients’ ovaries synthesize too much androgen, resulting in the recruitment of numerous pre-ovulatory small follicles. These follicles do not respond to the normal concentration of follicle stimulating hormone, and this hinders the formation of dominant follicles. In PCOS patients, the endometrium cannot be periodically shed due to the associated follicular development disorders and long-term lack of ovulation, and under estrogen stimulation the endometrium continues to thicken leading to atypical hyperplasia or even to carcinogenesis. A study conducted by Zhang et al. showed that about 97% of the enrolled PCOS patients did not ovulate, and 41% of them had endometrial hyperplasia, which suggested that endometrial hyperplasia in PCOS was induced by anovulation and continuous estrogen stimulation (Zhang et al., 2007). Han et al.’s meta-analysis showed that although most PCOS patients do not suffer from endometrial cancer, the morbidity of endometrial cancer in PCOS is still three times greater than that of healthy women (Hu et al., 2021). In the proliferative phase and secretory phase, the endometrium of PCOS patients is continuously exposed to estrogen, and the antagonistic effect of progesterone is weakened in the secretory phase. This pathological change likely results in reduced endometrial receptivity.

## HA and PCOS Endometrium

In the ovary, the production of steroid hormones is governed by gonadotropins and ovarian cell signaling. Androgen, a steroid hormone, converts to dehydroepiandrosterone (DHEA) and androstenedione in ovarian follicle cells and in the adrenal cortex through the activity of cholesterol side chain lyase (CYP11A; Nelson et al., 1999; Crespo et al., 2018; Sun et al., 2021). Approximately half of the testosterone a woman produces is synthesized by the ovaries and adrenal glands, and the rest is converted from the peripheral circulation (Horton et al., 1966; Kirschner and Bardin, 1972). The levels of circulating testosterone and androstenedione are elevated in PCOS patients (Franks, 1991), and more than 80% of PCOS patients have symptoms or signs of HA, such as hirsutism, acne, or alopecia (Sirmans and Pate, 2013). Studies have found that under circumstances of non-intervention or when using antagonists, such as gonadotropin-releasing hormone (GnRH), to inhibit the production of endogenous luteinizing hormone (LH), PCOS patients are more sensitive to exogenous LH (Gilling-Smith et al., 1997). Yazawa et al. isolated cells with the characteristics of hyper-secretion of steroid hormones from the follicular membranes of PCOS patients, which suggested that HA in PCOS patients is caused by more androgen being secreted by the theca cells (Yazawa et al., 2019). HA is an indispensable pathogenic factor for PCOS, and PCOS further aggravates the pathological state of HA, thus forming a vicious circle.

Androgen can inhibit the growth and differentiation of endometrial cells and the decidualization of the endometrium, thereby interfering with embryo implantation (Sanchez-Garrido and Tena-Sempere, 2020). Studies have shown that Wilms tumor protein (WT1), first discovered in nephridioblasts, regulates the proliferation and differentiation of embryonic and reproductive system cells, plays an important role in the process of endometrial decidualization, and regulates the expression of androgen receptors (Makrigiannakis et al., 2001; Anthony et al., 2003; Andersson et al., 2014). Gonzalez et al. took biopsies of the endometrium from infertile PCOS patients during ovulation and found that the elevated androgen in PCOS patients interfered with the regulatory balance between androgen receptor and WT1 compared with healthy patients (Gonzalez et al., 2012). WT1 is regarded as a specific and sensitive indicator of serous carcinomas of ovarian origin (Lizneva et al., 2016; Meczekalski et al., 2020), and studies have shown that the expression of WT1 in endometrial carcinoma is closely related to tumor hematopoiesis (Goodarzi et al., 2011).

Dehydroepiandrosterone, an adrenal androgen precursor, is involved in the synthesis of the androgen receptor agonists testosterone and dihydrotestosterone (DHT), and changes in DHEA can alter the bio-availability of endometrial androgen and the endometrial microenvironment. Gibson et al. found that DHEA can enhance the *in vitro* decidual response of human endometrial stromal fibroblasts (Gibson et al., 2018), and HA can also reduce the secretion of progesterone and endometrial receptivity by affecting the function of the corpus luteum (Brewer and Balen, 2010; Lee et al., 2020). Eagleson et al. found that androgen administration in women with normal ovulation can suppress serum LH levels and that long-term administration of androgen receptor antagonists in PCOS patients can ameliorate the negative feedback cycle (Cooney and Dokras, 2018). Meanwhile, HA can also cause cell cycle disorders in uterine tissue and can regulate cell death and survival pathways leading to endometrial hyperplasia. An animal experiment conducted by Ferreira et al. demonstrated that prenatal HA can disrupt the cell cycle in the uterus and can dysregulate cell death and survival pathways leading to uterine hyperplasia (Ferreira et al., 2020).

**Figure 2:**
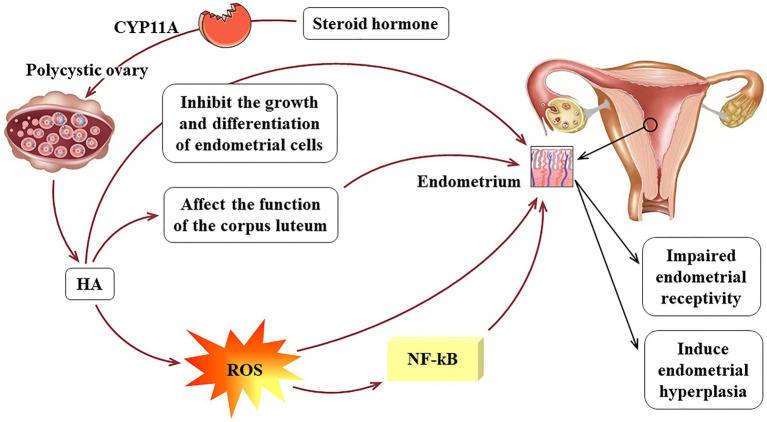
The pathological mechanism of HA acts on the endometrium of PCOS.

In addition, studies have shown that HA in PCOS patients stimulates oxidative stress as evidenced by the over-generation of reactive oxygen species (ROS; González et al., 2012). ROS can change the morphological and functional characteristics of endothelial cells, including permeability and adhesion molecule expression, leading to a continuous state of inflammation (Andrisani et al., 2014). Oner-Iyidoğan et al. found that compared with eutopic endometrium, hydrogen peroxide is increased in endometriotic cells, and the main effects of ROS on endometrial cells include oxidative damage and increased proliferation (Oner-Iyidoğan et al., 2004). Androgen can also induce the production of tumor necrosis factor-α (TNF-α) and interleukin (IL)-1, which promote the production of other inflammatory factors by binding to androgen-specific receptors, thereby activating the ROS system and the NF-kB inflammatory pathway. The NF-kB pathway has been shown to activate pro-inflammatory genes and to participate in the expression of molecular intermediates in human endometrium and first trimester decidua (King et al., 2001). A meta-analysis emphasized that HA can increase the risk of metabolic syndrome including IR and dyslipidemia (Chien et al., 2021). Although the relationship between HA and IR is mainly due to compensatory insulin secretion affecting the production of steroid hormones, too much androgen can reduce insulin sensitivity. In summary, HA inhibits the growth and differentiation of endometrial cells in patients with PCOS, which in turn reduces endometrial receptivity. In addition, high androgen levels can induce endometrial hyperplasia. The pathological mechanism of HA acting on the endometrium of PCOS is shown in Figure 2.

## IR and PCOS Endometrium

Insulin resistance occurs when the effect of insulin on cells, such as fat, muscle, and liver, is lower than normal, resulting in decreased glucose utilization, increased liver gluconeogenesis, and increased blood glucose concentration and compensatory hyperinsulinemia. Studies have shown that about 50–70% of PCOS patients also have IR and compensatory hyperinsulinemia, and the risk of type 2 diabetes is significantly increased in PCOS patients (Pani et al., 2020).

**Figure 3:**
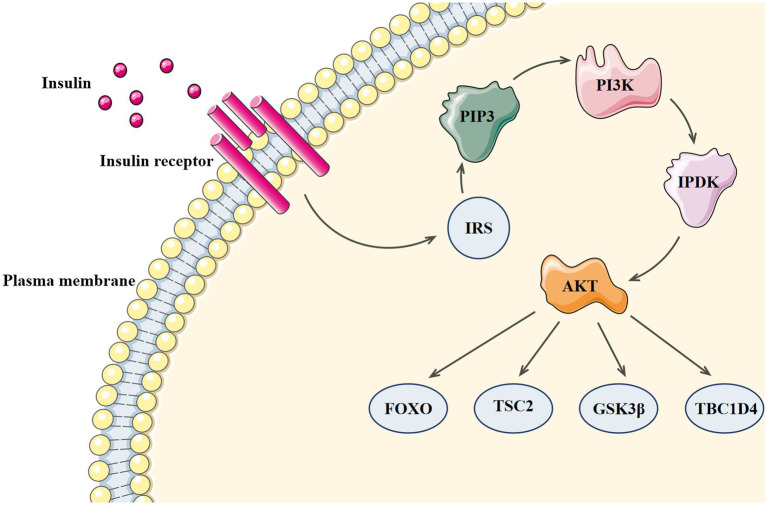
The PI3 Kinase-Akt signaling pathway.

Since the discovery of insulin in 1921, its mechanism of action has been widely debated (Smith, 2021). In 1985, Ullrich and Rutter’s team successfully cloned the cDNA of insulin receptor and developed the theory that tyrosine phosphorylation mediates intracellular receptor pathways (Ebina et al., 1985; Ullrich et al., 1985). Insulin receptor, a tetrameric adaptor protein consisting of two α-subunits and two β-subunits, belongs to the tyrosine kinase family. After insulin binds to the receptor, insulin receptor tyrosine kinase is activated and the conformation of the activated receptor changes, which increases the kinase activity necessary for substrate phosphorylation and causes tyrosine phosphorylation of insulin receptor and its substrate proteins. The phosphotyrosine sites on insulin receptor substrates allow lipid kinase PI3K to bind and recruit phosphoinositide-dependent kinase (PDK), which can directly phosphorylate Thr308 of protein kinase B (AKT; Stratiievska et al., 2018). Ser473 of AKT is phosphorylated for the second time by mTOR complex 2 (mTORC2), and the activated AKT continues to phosphorylate many substrates at Ser/Thr residues (including FOXO, TSC2, GSK3β, and TBC1D4) and to activate downstream effectors (Haeusler et al., 2018). The PI3 Kinase-Akt signaling pathway can be seen in Figure 3.

The mechanism of IR is complex and is related to obesity, metabolic abnormalities, low-grade inflammation, trace element deficiency, leptin resistance, etc. (Han et al., 2020; Jayaraman et al., 2021; Li et al., 2021). Studies have shown that IR may be involved in the signaling pathway after binding to the insulin receptor (Yang et al., 2021). Both the maximum glucose uptake rate and insulin-stimulated lipid suppression are reduced in the early stages of insulin signaling. Insulin, a negative regulator of its own signal transduction, downregulates the cell surface receptor when the insulin level increases, which contributes to reduced insulin signal transduction (Haeusler et al., 2018). *In vitro* studies have found that inhibition of serine/threonine phosphorylation can antagonize or terminate insulin signaling and thus is an important mechanism of IR (Sevillano et al., 2021). In addition, inflammatory cytokines and lipotoxicity can also inhibit phosphorylation (Kalafati et al., 2021; Lyu et al., 2021).

Related studies have shown that about 30% of PCOS patients with endometrial lesions also suffer from IR. By exploring the expression of molecules involved in the insulin pathway in the endometria from PCOS patients, Fornes et al. found that patients with hyperinsulinemia lack some of the insulin receptor substrates, which disrupts glucose metabolism in the endometrium and impairs endometrial receptivity (Fornes et al., 2010). Insulin inhibits the production of IGFBP-1, a biomarker of decidualization, suggesting that hyperinsulinemia can affect the normal function of the endometrium, leading to failed embryo implantation and increased abortion rate (Gupta et al., 2019). In addition, the endometrial inflammation environment triggered by IR may lead to progesterone resistance (Patel et al., 2017). The study conducted by Piltonen et al. found that the endometrial stromal fibroblasts in women with PCOS had an aberrant decidualization response to progesterone, indicating that this may be the cause of endometrial dysfunction, infertility, and pregnancy complications in women with PCOS (Piltonen et al., 2015). Epidemiological studies have shown that as early risk factors for endometrial hyperplasia, IR, and elevated insulin are significantly related to endometrial cancer (Fernandez-Montoli et al., 2021; Kliemann et al., 2021). The loss of PTEN expression may be an important early factor in endometrial carcinogenesis, and Yang et al. found that the loss of PTEN was reported in about 55% of endometrial cancer tissues, which may be related to the loss of mitotic function inhibited by insulin growth factor (Yang et al., 2015). In addition, IR can increase the sensitivity to local estrogen in the endometrium by reducing inflammation, and this promotes endometrial hyperplasia and carcinogenesis (Lv et al., 2019; Byrne et al., 2020). In conclusion, the abnormal endometrial microenvironment in PCOS patients with IR can lead to impaired endometrial receptivity, hyperplasia, and carcinogenesis.

## Progesterone Resistance and PCOS Endometrium

Progesterone is an important steroid hormone secreted by the corpus luteum of the ovary that regulates the entry of the endometrium into the secretory stage and ensures embryonic receptivity and thus plays an indispensable role in uterine embryo implantation, endometrial decidualization, and pregnancy stabilization (Bulmer and Lash, 1997; Monsivais et al., 2021). Progesterone normally plays a role in the “embryo implantation window,” but patients with PCOS have no progesterone effect due to long-term anovulation, which alters the periodicity of the endometrium. Progesterone resistance refers to the reduced responsiveness of target tissues to bioavailable progesterone (Chrousos et al., 1986; Al-Sabbagh et al., 2012), which can lead to the decline of estrogen’s antagonistic ability, the aggravation of inflammation, poor differentiation of the stroma, and obstruction of endometrial remodeling, and it is one of the major factors leading to the decline of endometrial receptivity and can significantly increase the risk of endometrial atypical hyperplasia and cancer (Savaris et al., 2011; Lessey and Kim, 2017; Simopoulou et al., 2021). Impaired progesterone response has been confirmed in the endometrium of patients with PCOS (Albaghdadi and Kan, 2021; Hamza et al., 2021), but the mechanism behind the effects of progesterone resistance in PCOS endometrium is not fully understood.

The expression of progesterone gradually increases in the early stage of hyperplasia, reaches its peak during ovulation, and then gradually decreases and disappears in the late stage of secretion, and this cyclic expression is a hallmark of endometrial differentiation and maturation (Li et al., 2014). The response of the endometrium to progesterone depends on the progesterone receptor (PR) in the nucleus, which consists mainly of the isomers including PRA and PRB (Pawar et al., 2015). Experiments have suggested that the antagonistic effect of progesterone on estrogen is mainly mediated by PRA (Kastner et al., 1990; Bulmer and Lash, 1997). The changed expression of PR and the activation and inhibition of related signal pathing pathways are the key pathways through which progesterone regulates endometrial function. An experiment carried out by Margarit et al. showed that the expression of PR in the endometrium of PCOS patients during the secretory phase was significantly increased compared with healthy women; the expression of PR in ovulatory PCOS patients was also significantly increased compared with anovulatory PCOS patients (Margarit et al., 2010). Hu et al. reported that the increased expression of PR in the endometrium of PCOS-like rats was consistent with the increased expression of estrogen receptor in PCOS-like rats (Hu et al., 2018). In PCOS patients, endometrial hyperplasia is often related to progesterone resistance, which may be caused by insufficient estrogen antagonism due to the low responsiveness of PR to progesterone (Hardiman et al., 2003; Shah et al., 2010).

Apart from the abnormal expression of PR in endometrium, the abnormal expression of related molecules downstream of PR pathway can also lead to endometrial progesterone resistance. For example, mitogen inducible gene 6 (Mig-6) is an important mediator of inhibiting the effect of estrogen in the progesterone signaling pathway (Yoo et al., 2015). Mig-6 is an important target of PR, and the over-expression of Mig-6 significantly enhances the pro-apoptotic, anti-proliferative, and anti-invasive effects of progesterone, suggesting that Mig-6 may be involved in progesterone’s inhibitory effect on estrogen in the endometrium and in the pathway of progesterone resistance (Yoo et al., 2018). The homeobox gene HOXA10, which is expressed in endometrial glandular epithelium and mesenchymal cells, is the regulatory target of progesterone on the endometrium during the menstrual cycle and is also an important intermediary for progesterone to play its role in endometrial receptivity. Cermik et al. have shown that HOXA10 is involved in the regulation of progesterone’s transcription targets and in the regulation of endometrial receptivity. The reduction of HOXA10 expression in the uterus might be one of the important reasons for progesterone resistance, poor endometrial receptivity, and reduced reproductive potential in patients with PCOS (He et al., 2018). In summary, progesterone resistance is an important mechanism leading to poor endometrial receptivity, endometrial dysplasia, and even endometrial cancer in patients with PCOS.

## Inflammatory Cytokines and PCOS Endometrium

The endometrium contains a variety of resident and transient immune cells, which together with cytokines and chemokines maintain the normal function of the endometrium (Strobel et al., 2021). Numerous studies have shown that chronic low-grade inflammation in PCOS patients is related to endometrial inflammatory cytokines, mainly including UNKs, C-reactive protein (CRP), TNF-α, and IL-6 (Liu et al., 2021).

Uterine natural killer cells, which are part of the endometrial cell population, not only promote embryo implantation, but also protect the embryo from pathogens (Trundley and Moffett, 2004; Dosiou and Giudice, 2005). UNKs are the most common endometrial leukocytes and are considered to be an endometrial marker of PCOS, and the number of UNKs peaks during the process of endometrial decidualization (Dosiou and Giudice, 2005; Piltonen, 2016; Bulmer and Lash, 2019). UNKs are associated with the synthesis of endometrial cytokines and can promote endometrial proliferation, differentiation, and repair by releasing inflammatory factors (Lyzikova et al., 2020). Studies have shown that endometrial inflammation in women with PCOS is intensified during the proliferative phase, and the concentration of UNKs decreases in the late secretory phase (Matteo et al., 2010; Piltonen et al., 2013). The number of UNKs in PCOS patients is significantly reduced during the secretory phase, which disturbs the effector functions of UNKs and ultimately leads to the loss of homeostasis of female reproductive tract homeostasis (Sala Elpidio et al., 2018).

C-reactive protein, an acute-phase protein mainly produced by hepatocytes, is a sensitive indicator of tissue injury and inflammatory response (Chen et al., 2017). Numerous studies have suggested that the level of CRP in PCOS patients is significantly higher than that in healthy women (Kalyan et al., 2018; Bannigida et al., 2020). In addition, it is the most reliable circulating marker of chronic low-grade inflammation in PCOS (Wang et al., 2017). Elevated CRP is usually positively correlated with IR and type 2 diabetes and is thus considered to be a potential cause of the long-term complications of PCOS (Marciniak et al., 2016). Socha et al. reviewed databases for meta-analyses, randomized controlled trials, and review articles looking to find possible mediators of carcinogenesis and cancer progression and concluded that CRP > 3.33 mg/L is related to the incidence of endometrial cancer with an HR = 2.29 (*p* < 0.05; Socha et al., 2021). The elevated concentration of CRP significantly increases the level of extracellular signal-regulated kinases, which can directly promote the proliferation and invasion of endometrial cells by activating the MAPK/ERK pathway.

Isolated endometrial cells from PCOS patients show altered gene expression, including IL-6 and TNF-α, indicating changes in the status of inflammation (Piltonen et al., 2013). TNF-α, a multifunctional pro-inflammatory cytokine, can promote the proliferation of endometrial cells, leading to increased levels of estrogen and its carcinogenic metabolites, which interfere with endometrial cycle exfoliation (Salama et al., 2009; Nair et al., 2013; Li et al., 2017). IL-6, a pleiotropic cytokine with roles in immunity and tissue regeneration, showed over-expression in PCOS patients compared with healthy controls (Alkhuriji et al., 2020). TNF-α and IL-6 could negatively influence insulin signaling through the interaction with insulin receptor substrate-1 (IRS1; Oróstica et al., 2020). TNF-α inhibits the IRS1 active form by promoting the phosphorylation of IRS1 at position Ser270. TNF-α also inhibits insulin signaling and changes insulin-induced glucose uptake by promoting the phosphorylation of members of the PI3K/AKT/mTOR signaling pathway (Abuelezz et al., 2020). IL-6 prevents the insulin receptor from interacting with IRS-1, thereby preventing the phosphorylation of IRS-1 tyrosine residues (the active form of IRS-1) and thus preventing the function of the insulin signaling pathway and thus blocking the PI3K/AKT pathway (Ozes et al., 2001). The pro-inflammatory cytokines in the endometrium can alter the natural path of action of insulin in PCOS patients through a variety of mechanisms leading to IR, disrupted glucose metabolism in the endometrium, and impaired receptor capacity. IR is highly related to chronic inflammation, and the two conditions influence each other in a vicious circle that disrupts the physiological endocrine and metabolic microenvironment of the endometrium and affects the receptivity of the endometrium (Patel, 2018).

## Conclusion

Clarifying the specific mechanisms (including gene loci and signaling pathways) of endometrial lesions caused by the various phenotypes of PCOS will provide new treatment strategies for improving clinical pregnancy outcomes in patients with PCOS and for reducing the risk of endometrial lesions. Future research should focus on screening more molecular targets of therapeutic drugs for PCOS and endometrium-related diseases.

## Author Contributions

YZ: funding acquisition. ZX, JL, and JF: conceptualization and writing – original draft preparation. HH, JinZ, and JiaZ: editing. YH, XW, and YZ: supervision. All authors contributed to the article and approved the submitted version.

## Funding

This work was supported by the National Natural Science Foundation of China (grant numbers 81774136 and 82074259), the Cultivation Project of the Outstanding Youth Fund of the Heilongjiang University of Chinese Medicine (grant number 2018jc02), and the “Outstanding Young Academic Leaders” Scientific research project of Heilongjiang University of Chinese Medicine to YZ.

## Conflict of Interest

The authors declare that the research was conducted in the absence of any commercial or financial relationships that could be construed as a potential conflict of interest.

## Publisher’s Note

All claims expressed in this article are solely those of the authors and do not necessarily represent those of their affiliated organizations, or those of the publisher, the editors and the reviewers. Any product that may be evaluated in this article, or claim that may be made by its manufacturer, is not guaranteed or endorsed by the publisher.
